# Supplementary material for: Factors associated with domestic violence in the Lahu hill tribe of northern Thailand: A cross-sectional study
Source: PLoS One. 2021 Mar 15;16(3):e0248587. doi: 10.1371/journal.pone.0248587 (PMC7959343; doi:10.1371/journal.pone.0248587)
Supplement: S1 Appendix — (PDF) [file pone.0248587.s001.pdf]

แบบสอบถามโครงการวิจัย

เรื่องลักษณะและผลกระทบของภัยเหล้ามือสองต่อสมาชิกครอบครัวในชนเผ่าลาหู่

ส่วนที่ 1 ข้อมูลทั่วไปของครอบครัว

1. จำนวนสมาชิกในครอบครัวทั้งหมด......................คน

2. อาศัยอยู่ด้วยกันจริงในปัจจุบัน ....คน

3. ประมาณการรายรับของครอบครัวต่อปี (ประมาณ).........................บาท/ปี

4.ความเพียงพอของรายได้ ☐ มีเงินเก็บ ☐ พอใช้ (ไม่เหลือเก็บ) ☐ มีหนี้สิน

5.มีที่ดินทำกินเป็นของตนเอง ☐ ไม่มี ☐ มี ระบุ .... ไร่

6. ในครอบครัวของท่านมีกลุ่มคนดังต่อไปนี้หรือไม่ (ตอบได้มากกว่า 1 ข้อ)

- เด็กที่อายุน้อยกว่าหรือเท่ากับ 15 ปี ☐ ไม่มี ☐ มี ....คน
- ผู้สูงอายุที่มีอายุ 60 ปีขึ้นไป ☐ ไม่มี ☐ มี ....คน
- ผู้ป่วยที่ไม่สามารถช่วยเหลือตัวเองได้ ☐ ไม่มี ☐ มี ....คน
- ผู้พิการ ☐ ไม่มี ☐ มี ระบุประเภท
  - ☐ คนพิการทางการมองเห็น ....คน
  - ☐ คนพิการทางจิตใจหรือพฤติกรรม ....คน
  - ☐ คนพิการทางกายหรือการเคลื่อนไหว ....คน
  - ☐ คนพิการทางสติปัญญาหรือการเรียนรู้ ....คน
  - ☐ คนพิการทางการได้ยินหรือการสื่อความหมาย.......คน

7. จากข้อ 6 ใครเป็นคนดูแลบุคคลดังกล่าว (ตอบได้มากกว่า 1 ข้อ)

☐ พ่อ

☐ แม่

☐ ลูก

☐ อื่น ๆ ระบุ..............................

ส่วนที่ 2 ข้อมูลส่วนบุคคลทั่วไป

1.บัตรประจำตัวประชาชนของท่าน

☐ บัตรประจำตัวประชาชนไทย (เลข13หลัก)

☐ บัตรประจำตัวคนซึ่งไม่มีสัญชาติไทย

☐ บัตรประจำตัวบุคคลที่ไม่มีสถานะทางทะเบียน

2. บทบาทในครอบครัวเมื่อเทียบกับหัวหน้าครอบครัว (☐ ผู้นำครอบครัว ☐ สมาชิกครอบครัว)

☐ ปู่ ☐ ย่า ☐ ตา ☐ ยาย ☐ ลุง ☐ ป้า ☐ น้า ☐ อา ☐ สามี (พ่อ) ☐ ภรรยา (แม่)

☐ พี่ ☐ น้อง ☐ ลูก ☐ หลาน ☐ ลูกสะใภ้ ☐ ลูกเขย ☐ อื่นๆ ระบุ.....................

3. เพศ ☐ ชาย ☐ หญิง

4. อายุ........................ปี

5. ลักษณะนิสัยของท่านปกติเป็นอย่างไร (ตอบได้มากกว่า 1 ข้อ)

☐ เก็บตัว ☐ อ่อนไหวง่าย ☐ ใจร้อน ☐ ใจเย็น ☐เชื่อมั่นในตัวเองสูง ☐อื่นๆ ระบุ

6. สถานภาพสมรส ☐ โสด ☐ คู่ ☐ หย่า ☐ คู่แต่แยกกันอยู่ ☐ หม้าย

7. ศาสนา ☐ พุทธ/ผีบรรพบุรุษ ☐ คริสต์ ☐ อิสลาม ☐ อื่น ๆ (ระบุ)..........................

8. การศึกษาขั้นสูงสุด/หรือชั้นที่กำลังศึกษา

☐ ไม่ได้เรียน ☐ ประถมศึกษา ☐ มัธยมศึกษา ☐ ปวช./ปวส./อนุปริญญา ☐ ปริญญาตรีหรือสูงกว่า

9. อาชีพ (ตอบได้มากกว่า 1 ข้อ)

☐ นักเรียน ☐ ว่างงาน ☐ เกษตรกร ☐ รับจ้าง ระบุ.....................

☐ ค้าขาย/ธุรกิจส่วนตัว ☐ ราชการ ☐ หาของป่า ☐ อื่น ๆ ระบุ...................

10.ลักษณะรายได้ที่ได้รับ ☐ ไม่มีรายได้ ☐ ได้ต่อเนื่องตลอดปี ☐ ได้เป็นบางช่วง (ช่วงที่ทำงาน)

11.มีโรคประจำตัวหรือไม่ ☐ ไม่มี

☐ มี ระบุ..

จำเป็นต้องไปพบแพทย์สม่ำเสมอหรือไม่ ☐ ไม่ใช่ ☐ใช่

12.ในช่วงหนึ่งปีที่ผ่านมาคุณได้พูดคุยกับคนในครอบครัวของคุณบ่อยเพียงไร

☐ทุกวัน/เกือบทุกวัน ☐ทุกอาทิตย์ ☐เดือนละ 1-3 ครั้ง

☐น้อยกว่า เดือนละครั้ง ☐ไม่เคยเลย

13.เมื่อมีปัญหาร้ายแรงคุณสามารถพึ่งพาคนในครอบครัวได้มากน้อยเพียงไร

☐ได้มาก ☐ได้บ้าง ☐ได้เล็กน้อย ☐ไม่ได้เลย

14.คุณสามารถเล่าเรื่องความกังวลให้คนในครอบครัวฟังได้มากน้อยเพียงไร

☐ได้มาก ☐ได้บ้าง ☐ได้เล็กน้อย ☐ไม่ได้เลย

15. คนในครอบครัวมีคนดื่มสุราไหม

☐มี ☐ไม่มี

16. ในหนึ่งปีที่ผ่านมาในครอบครัวมีปัญหาเรื่องเงินไม่พอใช้จ่ายไหม

☐มี ☐ไม่มี

ส่วนที่ 3 ประสบการณ์การดื่มสุรา สูบบุหรี่ และใช้สารเสพติด

**ในหนึ่งปีที่ผ่านมาท่านเคยใช้สารเสพติดต่อไปนี้หรือไม่ (การใช้นอกเหนือจากแพทย์สั่ง)**	**ไม่เคย**	**1-2 ครั้ง**	**เกือบทุกเดือน**	**เกือบทุกสัปดาห์**	**เกือบทุกวัน**
15.สูบบุหรี่	0	2	3	4	6
16.เหล้า เบียร์	0	2	3	4	6
17. กัญชา	0	2	3	4	6
18. ยาบ้า	0	2	3	4	6
19. กาว	0	2	3	4	6
22. ฝิ่น เฮโรอีน มอร์ฟีน เมทาโดน	0	2	3	4	6

ส่วนที่ 4 ประสบการณ์ที่ได้รับจากการกินเหล้ากินเบียร์ของสมาชิกในครอบครัว

24. ท่านเคยได้รับอุบัติเหตุ ในช่วงหนึ่งปีที่ผ่านมา โดยมีสาเหตุมาจากการกินเหล้า/กินเบียร์ของสมาชิกในครอบครัว หรือคนอื่นหรือไม่

ประเภทของอุบัติเหตุ	ไม่เคย	เคย	จำนวนครั้ง	หยุดงาน			นอนโรงพยาบาล			ความพิการ			เสียค่าใช้จ่ายต่อครั้ง (บาท)	ครั้งที่
				ไม่หยุด	1-7 วัน	มากกว่า 7 วัน	ไม่นอน	นอน	วัน	ไม่มี	ระบุความพิการ			
											ชั่วคราว	ถาวร		
1.หกล้ม														
2.รถล้ม/รถคว่ำ														
3.ขับรถชนสิ่งของ														
4.รถชนกัน														
5.อื่นๆ ระบุ.......														

25.ในหนึ่งปีที่ผ่านมาในครอบครัวของท่านมีการทะเลาะกันทางวาจา โดยมีสาเหตุมาจากการกินเหล้า/กินเบียร์ของสมาชิกในครอบครัวหรือไม่

☐ ไม่มี

☐ มี ☐ อย่างน้อย 1 ครั้งต่อสัปดาห์ ☐ อย่างน้อย 1 ครั้งต่อเดือน ☐ อย่างน้อย 1 ครั้งต่อปี

ใครเป็นผู้เริ่ม ☐ท่านเป็นผู้เริ่ม ☐ คนอื่นเป็นผู้เริ่ม ระบุ...

26.ในหนึ่งปีที่ผ่านมาในครอบครัวของท่านมีการทำลายสิ่งของ โดยมีสาเหตุมาจากการกินเหล้า/กินเบียร์ของสมาชิกในครอบครัวหรือไม่

☐ ไม่มี

☐ มี ☐ อย่างน้อย 1 ครั้งต่อสัปดาห์ ☐ อย่างน้อย 1 ครั้งต่อเดือน ☐ อย่างน้อย 1 ครั้งต่อปี

ใครเป็นผู้กระทำ ☐ ท่านเป็นผู้ทำ ☐ คนอื่นเป็นผู้ทำ ระบุ.................................

27.ในหนึ่งปีที่ผ่านมาในครอบครัวของท่านมีการทำร้ายร่างกายกันภายในครอบครัว โดยมีสาเหตุมาจากการกินเหล้า/กินเบียร์ของสมาชิกในครอบครัวหรือไม่

☐ ไม่มี

☐ มี ☐ อย่างน้อย 1 ครั้งต่อสัปดาห์ ☐ อย่างน้อย 1 ครั้งต่อเดือน ☐ อย่างน้อย 1 ครั้งต่อปี

ท่านเป็น ☐ ผู้ทำร้าย ☐ ถูกทำร้าย ☐ ผู้ทำร้ายและถูกทำร้าย

28.ในหนึ่งปีที่ผ่านมาสมาชิกในครอบครัวของท่านมีการทะเลาะกันทางวาจากับบุคคลภายนอกครอบครัว โดยมีสาเหตุมาจากการกินเหล้า/กินเบียร์ของสมาชิกในครอบครัวหรือไม่

☐ ไม่มี

☐ มี ☐ อย่างน้อย 1 ครั้งต่อสัปดาห์ ☐ อย่างน้อย 1 ครั้งต่อเดือน ☐ อย่างน้อย 1 ครั้งต่อปี

ใครเป็นผู้เริ่ม ☐ สมาชิกในครอบครัวของท่าน ☐ บุคคลภายนอกครอบครัว

29.ในหนึ่งปีที่ผ่านมาท่านมีการทำร้ายร่างกายบุคคลภายนอกครอบครัว โดยมีสาเหตุมาจากการกินเหล้า/กินเบียร์ของสมาชิกในครอบครัวหรือไม่

☐ ไม่มี

☐ มี ☐ อย่างน้อย 1 ครั้งต่อสัปดาห์ ☐ อย่างน้อย 1 ครั้งต่อเดือน ☐ อย่างน้อย 1 ครั้งต่อปี

ใครเป็นผู้เริ่ม ☐ สมาชิกในครอบครัวของท่าน ☐ บุคคลภายนอกครอบครัว

30. เมื่อท่านหรือสมาชิกในครอบครัวของท่าน เกิดความขัดแย้ง ทะเลาะ หรือการทำร้ายร่างกาย โดยมีสาเหตุมาจากการกินเหล้า/กินเบียร์ ท่านทำอย่างไร (ตอบได้มากกว่า 1 ข้อ)

☐เดินหนี หรือหลบออกจากสถานการณ์ความขัดแย้ง ☐พูดจาหว่านล้อมให้หยุดทะเลาะกัน

☐โต้ตอบด้วยคำพูดที่รุนแรง ☐เรียกให้คนอื่น ๆ มาช่วยห้าม

☐ตอบโต้ โดยการลงไม้ลงมือ ☐อื่น ๆ ระบุ.....................

ส่วนที่ 4.1 เฉพาะผู้หญิง

ในหนึ่งปีที่ผ่านมาท่านเคยประสบเหตุการณ์ดังต่อไปนี้ สืบเนื่องจากการกินเหล้า กินเบียร์ของสมาชิกในครอบครัวหรือไม่

ประสบการณ์	ความถี่ของการถูกกระทำ			
	ไม่เคย	น้อยกว่า 1 ครั้ง/เดือน	1-2 ครั้ง/เดือน	มากกว่าหรือเท่ากับ 3 ครั้ง/เดือน
1.การพูดจาส่อไปในทางเพศ พูดลวนลามทางเพศ				
2.การเปิดเผย หรืออวดอวัยวะเพศ ให้ดู				
3.ถูกกอดจูบ โดยที่ไม่เต็มใจ				
4.ถูกลวนลาม โดยการบีบ ลูบ คลำ มีการใช้นิ้วมือ แต่ไม่มีการสอดใสอวัยวะ				
5.พยายามหรือบังคับให้มีเพศสัมพันธ์				

6.พูดจาแสดงความหึงหวง				
7.ข่มขู่ว่าจะเลิก				

ส่วนที่ 4.2 เฉพาะคนที่อายุน้อยกว่า 15 ปี และ คนที่อายุ 60 ปี ขึ้นไป

ในหนึ่งปีที่ผ่านมาท่านเคยประสบเหตุการณ์ดังต่อไปนี้ สืบเนื่องจากการกินเหล้า กินเบียร์ของสมาชิกในครอบครัวหรือไม่

ประสบการณ์	ความถี่ของการถูกกระทำ			
	ไม่เคย	น้อยกว่า 1 ครั้ง/เดือน	1-2 ครั้ง/ เดือน	มากกว่าหรือเท่า กับ 3 ครั้ง/ เดือน
1.ถูกการทอดทิ้งให้อยู่ตามลำพัง				
2.ไม่ให้ค่าใช้จ่าย				
3.โยนภาระให้ดูแลบุตรหลาน/เหลน/ลูก /น้อง				
4.ไม่จัดเตรียมสิ่งจำเป็นพื้นฐานให้ เช่น อาหาร เสื้อผ้า ที่อยู่อาศัย การศึกษา ยารักษาโรค				
5.ไม่สนใจดูแลเมื่อเจ็บป่วย				

ส่วนที่ 4.3 สำหรับทุกคน

ในหนึ่งปีที่ผ่านมาท่านเคยประสบเหตุการณ์ดังต่อไปนี้ สืบเนื่องจากการกินเหล้า กินเบียร์ของสมาชิกในครอบครัวหรือไม่

ประสบการณ์	ความถี่ของการถูกกระทำ			
	ไม่เคย	น้อยกว่า 1 ครั้ง/เดือน	1-2 ครั้ง/ เดือน	มากกว่าหรือเท่า กับ 3 ครั้ง/เดือน
1.ถูกบังคับให้ไปซื้ออาหาร/เครื่องดื่ม/ถูกบังคับให้ทำงาน				
2.ถูกบังคับให้ดื่มเหล้า สูบบุหรี่ หรือสารเสพติดอื่น ๆ				
3.ถูกบังคับให้ไปขอ/ไปยืม/ไปแปะ เงินหรือสิ่งของ				

ประสบการณ์	ความถี่ของการถูกกระทำ			
	ไม่เคย	น้อยกว่า 1 ครั้ง/เดือน	1-2 ครั้ง/ เดือน	มากกว่าหรือเท่ากับ 3 ครั้ง/เดือน
4.พูดจาหยาบคาย/ดูถูก/พูดเยาะเย้ยคุณต่อหน้าคนอื่น				
5.แสดงอารมณ์โกรธ เหวี่ยง เกรี้ยวกราดเพื่อทำให้คุณกลัว				
6.ข่มขู่ว่าจะฆ่าตัวตาย				
7.ข่มขู่คุณว่าจะทำร้ายร่างกายของคุณ หรือทำร้ายคนอื่นในครอบครัว				
8.ข่มขู่ว่าจะฆ่าให้ตาย				
9.ผลักไสหรือไล่ให้ออกจากบ้าน				
10.ถูกผลัก ดึง ลาก กระชาก ขีดข่วน ถูกกัด ขว้างปาสิ่งของใส่				
11. ถูกตบหน้า/ต่อย/เตะ/ศอก/กระทืบ/บีบคอ				
12.ถูกทำร้ายร่างกายด้วยอาวุธ เช่น มีด ไม้ ปืน				
13.อื่น ๆ ระบุ..				

ส่วนที่ 5 เฉพาะผู้ที่ดื่มแอลกอฮอล์

1.ท่านดื่มแอลกอฮอล์ชนิดไหนเป็นประจำ (ตอบได้มากกว่า 1 ข้อ)

☐เบียร์ ☐ สุราขาว/สุรากลั่นชุมชน ☐ สุราสี/สุราแดง

☐สุราแช่พื้นบ้าน (สาโท, อุ, กระแช่) ☐ ไวน์องุ่น/แชมเปญ/ไวน์ผลไม้

☐ไวน์คูลเลอร์/สุราผสมน้ำผลไม้/เหล้าปั่น ☐ ยาดองเหล้า/สุราจีน/วอดก้า

2.โดยปกติท่านจะกินเหล้าหรือเบียร์ที่ไหน

☐ ร้านเหล้า/ผับ/บาร์/คาราโอเกะ ☐ ร้านอาหาร☐ หอพัก ☐ บ้านเพื่อน

☐ บ้านตนเอง ☐ ที่ทำงาน ☐ที่ประกอบศาสนา เช่น วัด/โบสถ์/ทำพิธีกรรม

☐ ซุ้มยาดอง/ร้านรถเข็น ☐สถานที่อื่น ๆ ระบุ..

3. แหล่งที่มาของเหล้าหรือเบียร์ (ตอบได้มากกว่า 1 ข้อ) ☐ซื้อเอง ☐ เพื่อนเลี้ยง

☐นายจ้างเลี้ยง ☐ อื่น ๆ.............................

4.โดยปกติท่านกินเหล้าหรือเบียร์ตอนไหน ☐เช้า ☐ กลางวัน ☐ ตอนเย็นหลังเลิกงาน

5.โดยปกติท่านกินเหล้าหรือเบียร์กับใคร

☐คนเดียว ☐ เพื่อนร่วมงาน ☐สามี/ภรรยา ☐ ลูก ☐ เพื่อน ☐ แฟน ☐ อื่น ๆ ระบุ....................

Audit Questions
