# Supplementary material for: Factors associated with domestic violence in the Lahu hill tribe of northern Thailand: A cross-sectional study
Source: PLoS One. 2021 Mar 15;16(3):e0248587. doi: 10.1371/journal.pone.0248587 (PMC7959343; doi:10.1371/journal.pone.0248587)
Supplement: S2 Appendix — (PDF) [file pone.0248587.s002.pdf]

Questionnaire

Part I General characteristic

1.Having Thai identification card with 13 digits

☐ Yes
☐ No and nothing
☐ No, other kind of card

2. Role in family

☐ Leader ☐ Family member

3. Sex ☐ Male ☐ Female

4. Age…………………years

6. Marital status ☐ Single ☐ Married ☐ Ever married

7. Religion ☐ Buddhist ☐ Christian ☐ Islam

8. Education

☐ Illiterate ☐ Primary ☐ Secondary ☐ Vocational school ☐ University

9. Occupation

☐ Student ☐ Unemployed ☐ Agriculturalist ☐ Employee
☐ Trader ☐ Officer ☐ Other

10.Income ☐ No ☐ Yes

11.Personal illness ☐ No
☐ Yes

12.How often do you speak with family member?

☐ Almost every day ☐ Weekly ☐ 1-3 times per month
☐ Less than one per month ☐ Long time

13.If you have a crisis in your life, how difficult to talk with your family member?

☐ Easy ☐ Somehow ☐ Only some ideas ☐ Very difficult

14.How difficult to talk with your family member while having worryness?

☐ Easy ☐ Somehow ☐ Only some ideas ☐ Very difficult

15. Do you have a family member who uses alcohol?

☐ Yes ☐ No

16. Did you have a financial problem in your family in the past year?

☐ Yes ☐ No

Part II History of substance use

**In the past year, do you use following stances**	**No**	**1-2 times**	**Monthly**	**Weekly**	**Every day**
1. Smoking					
2. Alcohol					
3. Marijuana					
4. Amphetamine					
5.Glue					
6.Opium					

Part III Women experiences in the past year from a family member who used alcohol

Experience	Yes	No
1. Sexual harassment		
2. Show off genital to see		
3. To be hugged and kissed by a woman without being willing		
4. Being harassed by squeezing and stroking using fingers without insertion		
5. Forced to have sexual intercourse		
6. Being spoken with jealousy		
7. Being threatened to separate		

Part IV Questions for children (aged ≤15 years) and elderly (aged ≥60 years)

Experience	Yes	No
1. Leaving to live alone		
2. Ignorance for financial support		
3. Leaving other people to take care		
4. Ignorance for providing essential items for life		
5. Ignorance to take care while having illness		

Part V Question for children (aged ≤15 years), women, and the elderly (aged ≥60 years)

Experiences	Yes	No
1. Forced to food and drink		
2. Forced to drink alcohol, smoke, or substance use		

Experiences	Yes	No
3. Forced to ask or loan money from other		
4. Expressing rude or insulting		
5. Expressing aggressively emotion to make you fear		
6. Expressing to kill themselves		
7.Expressing to hurt you		
8. Asking to kill others		
9. Getting kicked out of house		
10. Pushed, pull, scratch, or throw things		
11 Slapped, hit, kick, strangle		
12. Expressing to attack a people by weapon		
