# Supplementary material for: Factors associated with domestic violence in the Lahu hill tribe of northern Thailand: A cross-sectional study
Source: PLoS One. 2021 Mar 15;16(3):e0248587. doi: 10.1371/journal.pone.0248587 (PMC7959343; doi:10.1371/journal.pone.0248587)
Supplement: Attachment — Submitted filename: Response go reviewers.docx [file pone.0248587.s004.docx]

**Response go reviewers’ comments**

Dear Editor,

We have used long time in re-anaysis and re-forming the presentation, checking in whole text and response to all comments of reviewers. Some comments, we could not response because there is no information available. However, we very hope that you and reviewers would be happy in this version.

Finally, we sorry that using a longer time to be able to response to your comments.

Best regards,

TK

Editor comment

We have received comments from three reviewers. One reviewer recommended rejection, one recommended minor revisions, and the other one recommended major revisions. The primary concerns are around the sampling and data analysis methods. I agree with two reviewers that stratified analyses should have been performed to explore the differences in domestic violence prevalence and its risk factors in different sub-populations. The sub-populations included girls and boys under 15 years, women and men, and female and male elderly over 60 years. The authors expressed an interest in studying the subgroups and should have reflected this in the analyses. The lack of sub-group results introduced a challenge in discussing and comparing this study's findings against other studies. We will consider this manuscript for further review if the authors could address these concerns.

1.We suggest you thoroughly copyedit your manuscript for language usage, spelling, and grammar. If you do not know anyone who can help you do this, you may wish to consider employing a professional scientific editing service.

: Thank you very much for the points raised, we have a lot concern about that. The manuscript is checked and re-edited by the American Journal Experts.

2. Please include a copy of the questionnaire, in the original language, as Supporting Information.

: Thank you, it’s attached (Thai version).

3. In statistical methods, please clarify whether you corrected for multiple comparisons.

: We revised the whole section of data analysis. There are divided into three groups; children (5-15 years), women (aged 16-59 years), and elderly (aged 60 years and over).

All steps of the analysis, we sued the multiple logistic regression to compare between who had violence and did not to fine the final model before interpreting the factors associated with domestic violence in each group.

4. Cross-sectional study design cannot be used to determine causation. In this light, please revise your conclusions to indicate that your study presents associations between exposure variables and domestic violence. For example, your statement that "smoking and alcohol and amphetamine use, leads to domestic violence" implies causation which is cannot be determined using your study design.

: Thank you very much for such great comment. All points have been revised including the mathematic forms used in abstract, results, and discussion (page 2; lines 14-24; page 11, lines 7-15.

5. Please amend your list of authors on the manuscript to ensure that each author is linked to an affiliation. Authors’ affiliations should reflect the institution where the work was done (if authors moved subsequently, you can also list the new affiliation stating “current affiliation:….” as necessary).

: Thank you, we have done in double checked.

6. Your ethics statement should only appear in the Methods section of your manuscript. If your ethics statement is written in any section besides the Methods, please move it to the Methods section and delete it from any other section. Please ensure that your ethics statement is included in your manuscript, as the ethics statement entered into the online submission form will not be published alongside your manuscript.

: Thank you so much, it has been moved into the proper place (page 6, line 10-16). All information in the ethical statement is the same put into the system during submission the manuscript.

8. Please include captions for your Supporting Information files at the end of your manuscript, and update any in-text citations to match accordingly. Please see our Supporting Information guidelines for more information: <http://journals.plos.org/plosone/s/supporting-information>.

: Thank you very much for the suggestion. All relevant supporting information have been placed in a proper place in the manuscript with corresponding to the journal guideline.

Reviewer #1: Thank you for the opportunity to review “Factors associated with domestic violence in the Lahu hill tribe of northern Thailand: A cross-sectional study.” The authors have tackled an important public health issue ie violence against the 3 vulnerable groups. However, I have several pertinent issues of concern with the study/paper, which I will outline below.
i) One of the main concern the study methodology and analysis procedure. The author indicated in their methods section of the paper that the target populations of interest were children (<=15 yrs), women and the elderly. They have also outlined the questionnaire items used to measure violence in each of the 3 groups. The authors then went on to derived a measure for domestic violence from these items. Considering that the drivers of violence in the 3 groups are likely to be different, I would have preferred if the authors conducted a stratified analysis instead so they can look at these groups separately. The type of violence experienced by the 3 groups are different and most likely perpetrated by different members of the HH. Possibly the factors associated with violence experienced by the 3 groups are also different.

: Thank you so much for this valuable comment. We totally agree with you and then making different procedure for the analysis (three different group, three stratifies).

: The results show very interestingly in table 4 (violence to women), table 5 (violence to children), and table 6 (violence to the elderly)

ii) Another concern is the study methodology. The authors have not clearly outlined how participants from households were recruited. Did they include everyone in the household (children, mother & father and the elderly)? If they did, did they account for clustering by household in their analysis? If they did not, then they need to clearly explain in their methodology how HH members were selected.

: All eligible populations (Children aged 5-15 years, women aged 16-59 years, and elderly 60 years and above) who live in 20 selected Lahu villages were invited to the study.

iii) The authors have defined children as <=15yrs. They need to clearly indicate the minimum age for this group in the methods section (as part of inclusion criteria definition) and how data was collected from them (eg was it interviewer administered?). I understand in Table 1 they have outlined that the minimum age in the sample was 5yrs. But it is not clear whether 5yrs was a cut off or just minimum age for those interviewed. I am also rather concerned about the integrity of information on violence corrected from children (10yrs or less)

: We intended to collect data from children 5-15 years, which come from our 13 years experience in doing research among the hill tribe. Below 5 years is very difficult to obtain the validate information.

iv) The authors also indicated in the methods section that the target population of interest were children (<=15yrs), women(16-59yrs) and the elderly (>=60yrs). However, their data shows that they also have men (n=382) aged between 16-59yrs in the sample. I do not understand the rationale for including men (16-59yrs) in the study.

: We so sorry for the mistake of process of case selection during the analysis. We have mad revised in whole process of data analysis.

v) The general norm for violence studies is to analyze data for male and females separately as they tend to have different risk factors. I would prefer that they present their analysis that way.

: Thank you very much for the comment. We have now making three subcategories for the analysis; children aged 5-15 years, women 16-59 years, and the elderly aged 60 years and over. The whole characteristics and other information have been separated.

Other general comments are:
• They can make their tables more succinct by reporting one category for all binary variables (‘yes”) rather both ‘yes’ and ‘no’

: Thank you for the comment, we have tried to make the best option, and present in Yes and No to confirm the readers on the validation of the presentation. We very hope you understand us.

• The manuscript can do with some proof reading and editing.

: Thank you so much, it was re-edited by the American Journal Experts.

• The author should consider having prevalence estimates for each type of violence (eg physical violence).

: Yes, the data are presented in the results section.

• With the study having 3 groups, it is not clear if participants were reporting their own behavior ( eg alcohol use) and wondering what could be the impact of these on the estimates.

: We have classified all items or factors according to three groups, in table 2. Very hope it helps.

• Sample size formula (if it is necessary to show it) should be explained in the context of the study, e.g what is p in this study?

: Thank you, we have carefully explain detail, please see page 4-5.

• They could have a separate table for the multivariate analysis.

: Yes, we did. There are now separated into different table, table 4 (children), 5 (women), and 6(elderly)

Reviewer #2: Background
1.Please clarify the scope of this study because there is different definition between domestic violence and intimate partner violence so the reader might more clear and understand (Domestic violence is violence in the family, the perpetrator is family member, whereas intimate partner violence is violence between partner)

: Yes, we intended to understand the domestic violence which is defined as a pattern of behavior in any relationship that is used go gain or maintain power and control over a family members. This definition is followed the UN definition of domestic violence. Page 6.

2. The authors should mention research gaps and why have to conduct this study in Lahu tribe.

: We did, page 4, lines 7-10.

Method
1. How the researchers randomly selected the village?

: We did selection the village by which were selected by a computer generate random number method, page 4, lines 17-18.

2. The sample size calculation is n=826 but the researcher collected the data from n=1,028. Please calrify and give the explanation

: We so sorry, some procedure in sample size calculation was incorrect. We have made correction, page 4 line 27-31, and page 5, line 1-5.

3. What is the reliability (pre-test) value from this study?

: The overall Cronbach alpha was 0.71, page 6, line 4.

Result
1. Heading of Table 3 show n= 647 but in detail in the table showed n= 1,028. However, some item "n" is lower than 1,028. Please check the consistency of the data

: So sorry for the mistake, all data in all table have been double checked and revised.

2. Recoomendation pls consider to give recommendation to 3 level; individual, community, and policy level

: We have added in conclusion section, page 28, lines 12-25.

Reviewer #3: General comments: Authors have made a good attempt to analyse the extent of domestic violence and factors associated with domestic among children, women and the elderly in a poor community in rural Thailand. This paper will contribute to understanding of domestic violence in low and middle income countries, and provide data for understanding subgroups. However, the authors have introduced interest in studying subgroups but have not done so in the analyses, and this presents a challenge in the analysis and discussion of the generalize results as they often seek to make comparisons or justifications of their results against subgroups elsewhere without actual analyses of their own to back up the comparisons. My recommendations are that the authors must revise their analyses and present the overall study population analysis and present children: girls and boys under 15 years, women and men (by whichever is legal age limit for marriage perhaps), elderly women and men over 60 years. The lack of data on perpetrators is a major limitation but inferences may be safely drawn with supporting study as to who the common perpetrators of DV usually are in such contexts in Thailand. The subgroup analyses will help authors make a case for the understanding of how substance abuse increases risk of violence for specific subgroups to some extent even without understanding the perpetrators data.

: Thank you so much, we have completely done on classifying to analysis the factors associated with three different groups; children (table 4), women (table 5), and elderly (table 6)

Abstract:
Revise according to the recommendations from the content of the manuscript

: Thank you, it’s revised and improved.

Background:
“Lead to”: Authors may not reach conclusion that certain variable ‘lead to’ certain risks with cross-sectional data including their own study as such studies only allows for determination of risk factors. We do speak of associations unless experimental studies were done.

: Thank you, we have changed as comment.

“Substance use” versus abuse: authors have described substance ‘use’ and not abuse this makes it difficult to determine risk outright without measuring the extent of misuse.

: Thank you, it’s changed properly in whole text.

The background starts well with the global picture but needs to locate the domestic violence problem and risk factors in the context of Thailand, and do so indicating the extent to which other studies have addressed this issue.

: Thank you,

: We have revised and mentioned many risk factors related to domestic violence in Thailand and its references, page 3, line 20-29

- Go into some detail about the Thai social life, income, earnings, religion, culture and describing who Thai people are, what other tribes or social groupings exist in Thailand and the extent of violence there as well as any risk factors or protective factors against DV.

: We have greatly investigated on the information between Thailand and the hill tribe, unfortunately there is little information found, and no information presented in protective factor. We would say that no-study had been done previously about domestic violence in the hill tribe. So sorry.

- Also contrast with other neighbouring countries where possible, at least in terms of the prevalence.

: We have explored the information, unfortunately there is no scientific information regard to domestic violence among tribe people in Myanmar or Laos as well.

: Information is presented to non-scientific writer which is mostly not relevant to domestic violence.

- Any cross-border issues that make the hill tribes different from other Thai society?

: The different of the hill tribe and Thai people is not from cross-border but rather from the difference of their culture and other life styles.

- Describe the context of substance use and substance abuse in Thailand. How are amphetamines accessed? Natural or other synthetic stimulants? History of substance abuse. What kind of alcohol is used or abused? How is it sourced in communities which have been described as poor? How do they access amphetamines?

: We have added in reference 16-17. Even Thailand has been reported on having amphetamine use, but surprising there is very few scientific papers available.

- I also propose that some effort into explaining violence against children younger than 15 years be made, and explaining how such studies were done in Thailand or neighbouring countries. What are the risk factors?

: We have added some information in the discussion part. However, there is very few paper published.

Page 3:

Paragraph 1:
- the mention of the SDGs: This is a goal and not a fact, so the authors need to talk about this SDG in the correct context. What are authors trying to say about this goal?
- at the end of paragraph: What do the authors mean ‘domestically’?

: Thank you very much for the great comment.

Paragraph 2:
- at the end of the paragraph, authors mention contributors to violence: Do authors mean ‘perpetrators’? how do they contribute? If as the perpetrators then authors should state that explicitly. And mention other contributors if they exist. In some context, we have instigators and some Asian studies refer to instigators.

: We are trying to say that under the context of Thai society which men dominate in family, including the referred paper said that the men was defined as the domestic makers.

: We sorry if the response is not going to what your questions. If you could make more easier would be great appreciated.

Last paragraph describing hill tribes needs to go into the Setting/study site information in the methods section and explain the following: What is the population size relative to the largest and smallest tribe? Describe the lifestyle that distinguishes Lahu people from others?

: Thank you for the comment. We studied in the Lahu because one of our small previous study presented that their have a large proportion who used alcohol. Moreover, some information were found on the domestic violence among the Lahu people. This become our current study. Unfortunately, we do not have information about very some sensitive behaviors such as substances use including domestic violence in other tribes. Then, we decide not to mention the information of other tribes. We so sorry, we will do more study before could be able to come up the scientific information these points.

Last paragraph of the Background section: Provide a justification for the paper explaining the conceptual framework used for this study as mentioned in the methods section. Thus explaining why a paper on prevalence and associated factors. Why was the study conducted? And why its important for the paper to be published?

: Thank you so much for such great comments here.

: We have modified, improved, and revised as your comments. Page 4, lines 7-10.

Methods:
Paragraph 1 - Explain size of the Lahu hill area. Sources of living such as access to water, income, who is working or not, what is the family structure?

: Thank you, it’s revised in whole paragraph. Page 4-5.

Paragraph 2 - Study sample recruitment needs to be explained. The study aim suggests data was collected for children, women and older persons. It is not clear though what the sampling unit was for the study. Was it families or just individuals in the community?

: Thank you, it’s revised and explained in page 6, line 14-28.

Paragraph 4 regarding the questionnaire:
- Which questions were inappropriate for the Lahu people due to culture and beliefs?

: Do you kick your wife? In this question, we can ask directly. Many questions, they did not answer if they did not trust interviewer.

: Do you think you have a problem with your husband in amphetamine use?

- Last sentence about pilot is incomplete. Explaining the pilot is good. So finish the sentence please.

: Thank you and sorry for the mistake. It’s improved.

Paragraph 5 about the questionnaire:
- Authors need to present a table of each set of questions measured, describing what the variable is made up of, and explaining the source of the variables especially if used in other studies or new including those verified and changed with inputs from the Lahu people.

: We used our own questionnaire developed. The detail is explained in page 5 and 6

: The detail of questionnaire both Thai and English have been attached.

- Please name that dependent variable and clarify what it comprised of it is a combination of variables. What is the outcome variable made up of?

: DV is domestic violence which is very special forms occurred in Lahu family, see page 6, lines 4-12.

: We have known the forms from our pilot step. All forms have been developed to questions which present the result in Table 3.

- Domestic violence questions need to be explained in more detail as an outcome variable. The explanation given here is different compared to what is presented in the results. Clearly explain all variables here first, what they entail including any variation that may be presented in the results. Also provide the time frame that is reported for each form of DV, lifetime experience or in the past 12 months?

: It’s improved and explained in page 6, line 5-12.

Paragraph 6:
- Informed consent forms are meant to remain with the authors particularly to demonstrate that consent was sought and obtained from participants. Informed consent is also a process, it is not clear how this was done. Rather explain how information about the study was provided to participants, what was were these essential aspects that were explained, and how consent was given by particpants.

: Thank you, it’s explained in page 6, lines 11-16.

- Sentence: “People who were not able to provide essential information due to personal health problems were excluded from the study” does not explain the eligibility criteria for sample to participate in the study. Please explain the criteria used, was it one factor or multiple factors that were considered?

: Thank you, it’s explained. Page 4, lines 24-25.

- Issue of language used by participants: Move this to the questionnaire section. Was this a paper-based questionnaire? Explain how data was collected in more precisely, like language, who collected the data, how they were trained, their level of qualification. What the role of the village health volunteers including what they usually do.

: Thank you, we were helped from the village health volunteers in filling general information. However, those who could not read Thai, the forms were filled by psychiatric nurse with the help of community heath volunteers,

- How was a confidential room determined? Maybe explain this in a different way? Merge the note about ethical clearance at the end of this paragraph.
Data analysis:

: A confident room is provided by village headmen at the community hall. Page 6, line 22-23.

Logistic regression modelling is a process, explain that process. What was the outcome variable, candidate variable, how they were determined first, were bivariate analyses performed first, how were candidate variables selected and fitted into the model, any process of elimination engaged? This is crucial information to determine what was done and whether it can be replicated and reach the same conclusions the authors did.
: The DV is the having domestic violence (Y/N). Then, it forces us to us the logistic regression for the analyses. The stepe of the analysis, we used “Enter” mode which is allowed researcher to consider a IV put into the model because we have to concern on statistic significant and public health significant.

: Detail have been explained in page 6, line 30-31, and page 7 lines, 1-4.

Results section:
- Table 1 and Table 2 and Table 3 are difficult to decipher because the study is looking at 3 sets of participants: children under 15, women, older persons older than 60.

: Thank you, we have revised in analyses and interpretations in whole set of data. Please see table 1-6 which are classified into three main groups.

- Authors need to demonstrate the same characteristics for the study population alongside these characteristics for the study population sets. Including data from men and boys and classifying elders by difference between women, men, girls, boys, older women, older men. The socio-demographics do not make sense without a clear understanding of what the univariate data looks like for all sets of individuals. This is important as data also needs to account for any child marriage which is possible under 15 years, number of family members living in the same household, etc. Vulnerability to violence is also determined by gender. Cross tabulation of the differences by gender is also key in this analysis and table.

: We agree with you, and did reanalysis in the whole set of data, please see table 1, page 7.

- In Table 2 there are other drugs that have been measured which are not explained in the methods section. This justifies why a table including types of substances is needed.

: From our pilot stage, most of substances use among the Lahu had been clarify, then we intended to measure only this 6 items.

- Table 3: Authors can provide an overall estimation of DV for the population of interest, but still need to extent the analysis to demonstrate the differences between children, women and elderly persons, so that there is clarity on the extent of vulnerability in the population including their subgroups, this is most useful to provide guidance to which groups health interventions should prioritise and address and what actual interventions could be considered for which subgroups or to address which factors associated with vulnerability of which subgroup. Lumping all children, women and elderly persons is not justified as an end in itself, but would be more valuable if authors are able to single out the most vulnerable, even at a comparative level. The analysis is insufficient at this level and needs to demonstrate the complexities that are entailed in domestic violence as a multi-layered construct that can be differentiated for women into intimate partner violence or domestic violence by family members other than husband if women are married, or child abuse by parents or other family members, or even strangers if the latter was measured, etc.

: Defiantly, we have revised the analysis in table 3, please see pages 11-12.

- In Table 3, for each type of domestic violence need to provide a composite estimate as done with domestic violence. This will explain the extent of physical or emotional abuse overall.

: Thank you, it’s revised and improved.

- Authors need to explain the risk of smoking and how it links to perpetration of violence, as this variable doesn’t make sense in terms of how it increases vulnerability to DV. Do this in the background section.

: We have modified the discussion in this part and added some information. Page 26, lines 1-12.

- Table 4: The analysis appears to have skipped a stage as not all variables need to go into the multivariate analysis, so authors need to demonstrate candidate variables through bivariate analysis and look at some degree of association of each variable with the outcome variable. So bivariate analysis is required. Authors much explain Why are some analyses adjusted for and others are not?

: Sorry, please forget the old style of the analysis and presentations. We did re-analysis in classifying into three groups, please kindly see table 4-6 in new results.

Discussion:
- The authors are evidently struggling to explain the study prevalence in it entirety without a clear indication per children, women, elderly women or men subsets analysed. The data does not provide data to indicate the extent of violence against children. The story of substance use in general is also not telling a clear story without understanding the extent to which increased risk and perhaps authors should also report on who is using, and whether combinations of substances are used and determine risk from that angle. However, the conclusions reached in this paper cannot be reached with the currently presented analyses. Authors need to address the analytical questions about extent of vulnerability to violence per subgroup, what increases their vulnerability in most precise terms than the general picture painted already which does not in itself make much sense. Then tell the role substances play in this story.

: Thank you, you are right. We have made revision in whole paragraph after getting information from the analysis in three groups.

- Paragraph 2: These comparisons are incorrect without a clear understanding of the prevalence of DV among women from your analysis, so this is incorrect comparison. The same point applies to comparisons on data about children or the elderly. The analyses failed to demonstrate prevalence for each subgroup so there cannot be comparisons with subgroups.

: Yes, we agree with you. We have revised in this whole paragraph after getting the three groups analyses.

- Paragraph 3: This is suggesting that Buddist society is more gender equitable, where is the data to support this? The argument does not make sense as patriarchy is a common factor across all societies. Authors need to provide a clear explanation of the religious differences from the background and link their interpretation to that. It may be that there is a vast difference based on religions but the current argument to explain these results is incorrect without studies to support it. Authors need to explain what they mean by ‘complicated relationships’. How is Buddhism organized and are relationships there complicated too or not?

: Unfortunately, after carefully re-analysis in three different groups, religion was not found the association with domestic violent. Then, it’s deleted from the sdicussion.

Conclusions:
The conclusions need to be strengthened and be drawing out what the results have demonstrated about the study sample as sub-groups and as a whole, and indicating more specifically what recommendations suite which aspect of the results.

: Thank you, It’s revised and improved.

Abbreviations:
Authors must integrate in the texts and ensure each is explained in full at first mention and then abbreviated afterwards.

: Thank you, we have checked all relevant text.

Ethical considerations
- must be integrated with the details on how consent was obtained and data collected. Add a little bit on what DV specific considerations were made? What guidelines were followed for conducting these interviews on such a sensitive subject? Explain data storage.

: Thank you for the comment, page 6, lines 4-12.

: the ethical has been moved to methods section. The content is also extended.

References:
Many of the references do not follow the journal’s preferred referencing style. Visit: <https://journals.plos.org/plospathogens/s/submission-guidelines#loc-references> for information on Vancouver style. Here is some simpler information about what you can do and examples provided.
PLOS uses the reference style outlined by the International Committee of Medical Journal Editors (ICMJE), also referred to as the “Vancouver” style. Example formats are listed below. Additional examples are in the ICMJE sample references. A reference management tool, EndNote, offers a current style file that can assist you with the formatting of your references. If you have problems with any reference management program, please contact the source company's technical support.

: Thank you so much, all references have been re-checked and re-formatting.
