# Supplementary material for: Factors associated with domestic violence in the Lahu hill tribe of northern Thailand: A cross-sectional study
Source: PLoS One. 2021 Mar 15;16(3):e0248587. doi: 10.1371/journal.pone.0248587 (PMC7959343; doi:10.1371/journal.pone.0248587)
Supplement: Attachment — Submitted filename: Response_to_editors_Harms_FINAL.docx [file pone.0248587.s005.docx]

**Response to Editor’s comments**

**Dear Editor,**

I do not know how to thank you for the help along the paper. I have learnt a lot from your comments in each item. Your advices comments make us improvement all skills and practices in how to write a good academic paper.

After improvement, the paper has been once again checked by the American Journal Experts.

I do very hope that you happy in this version.

Thank you once again,

TK

Additional Editor Comments:

**Editor’s comments**

We thank the authors for addressing outstanding comments from the reviewers. The revised manuscript has been improved. However, the English writing quality remains well below an acceptable level, particularly. I have read the entire manuscript; however, my comments are not exhaustive. The manuscript requires substantial support from an experienced English writer with sufficient understanding of the research context.

: Thank you very much for your comments. The manuscript has been re-edited by American Journal Experts, with reference. no 3987-18F0-6D5E-AB8E-72FF .

In your response to reviewers, please provide the details on how you addressed the comments (what changes were made, where in the text are revised), not just saying those particular points were revised and improved (but what were revised or improved and where). Where data were reanalyzed, explain briefly the new findings and ensure that revisions have been made to reflect the new findings (what were added or removed and where). To most of the comments, particularly those from Reviewer #3, the responses are not sufficient without clear explanations. In your revised manuscript preparation, please have a closer look at the Instructions for Authors and follow them strictly, including word count limit, styles, spacing adjustments, etc.

**Abstract**

1. The abstract must be condensed as it contains far more than the recommended 350 word count limit.

: The abstract has been revised to 328 words.

2. Line 26, page 1: Please remove ‘sexual problems’ as it is outside of the study’s scope and is not applicable to all target populations under the study (e.g., children).

: Thank you. This term has been deleted.

3. Please use consistent terminologies; e.g., ‘the elderly’ in place of ‘elderly,’ ‘elderly adults,’ ‘elderly individuals,’ ‘older people,’ ‘elderly persons,’ etc. throughout the text.

: Thank you. This terminology has been replaced in the entire text.

4. Line 26, page 1: ‘types of’ is unnecessary.

: Thank you. This phrasing has been improved.

5. Line 30, page 1: The word ‘adults’ is unnecessary.

: Thank you. This term has been removed.

6. Line 1, page 2: A cross-sectional study was conducted among participants who belonged to the Lahu hill tribe and lived in 20 selected villages in Chiang Rai Province, Thailand.

: Thank you. This has been changed.

7. Lines 2-5: A validated questionnaire was used to collect personal information and experiences related to domestic violence in the past year from children aged 5-15 years, women (aged 16-59 years), and elderly aged 60 years and over.

: Thank you. This has been changed.

8. Line 5, page 2: Please specify whether the ‘logistic regression analyses’ were bivariate, multiple, or both.

: A binary logistic regression was used. The wording has been added.

9. Line 6, page 2: Please remove ‘at the significance level µ=0.05.’

: Thank you. This has been deleted.

10. Results section must be shortened, removing most of the descriptive socio-demographic results.

: Thank you. This has been deleted. Moreover, the abstract has been modified to meet the 350-word limit.

11. The word ‘analysis’ is a countable noun, and its plural form should be ‘analyses.’

: Thank you. However, we changed the word to “in the study”.

12. Line 10, page 2: The use of ‘while’ is incorrect.

: Thank you. This wording has been improved.

13. Line 12, page 2: …children who smoked…

: Thank you. This has been deleted.

14. Line 15, page 2: …a family head…

: Thank you. This has been changed.

15. Lines 14-20, page 2: The large sentence must be restructured as it is difficult to read. It may be broken down into small sentences. Also, when you present the associations between variables, please make the significance level clear, not just say ‘more likely.’

: This has been revised.

**Introduction**

16. Line 7, page 3: In scientific writing, numbers smaller than 10 should be spelt out.

: Thank you. This has been corrected.

17. Line 19, page 3: …the elderly living in developing countries…

: Thank you. This has been improved.

18. Lines 21-23, page 3: Please revise the sentence to minimize wording redundancies (children, women, the elderly) and improve the referencing. The references 11, 12, and 13 should be moved to join 14 and 15 at the end of the sentence.

: Thank you. This has been improved.

19. Line 24, page 3: Please remove ‘who were’ as it is unnecessary.

: Thank you. This has been improved.

20. Lines 26-27, page 3: “Given people’s economic constraints and substance use, alcohol use has become an integrated factor contributing to domestic violence [18].” This sentence is difficult to understand – how is the first part linked to the latter?

: Thank you. This has been revised and improved.

21. Line 8, page 4: Add ‘,’ before ‘including.’

: Thank you. This has been improved.

**Methods**

22. Lines 16-17, page 4: The first and second paragraph should be combined as the first one does not make much sense. Consider: “A cross-sectional study was conducted in 20 villages randomly selected out of 216 Lahu villages in Chiang Rai Province, Thailand [21], using a random number generation method.”

: Thank you very much for the help. The paragraphs have been accordingly revised and improved.

23. Lines 18-19, page 4: Please remove “However, in the study process, only 20 Lahu villages were randomly selected from the list” as does not add any meaning to the text and sounds redundant.

: Thank you. This has been removed.

24. Lines 19-20, page 4: The sentence, “All the Lahu people who were living in the selected 20 villages were invited to participate in the study” is not true as you included only children aged 5-15, women, and the elderly.

: You are correct, and this sentence should not appear in this section. We have modified it and moved it to the next section.

25. Line 22, page 4: Again, the same sentence was repeated, which should also be removed.

: Thank you for the comment. As we removed the sentence in response to comment no. 24, we need to maintain the sentence in this section.

26. Lines 24-25, page 4: The sentence “People who were able to provide essential information regarding the study protocol, living selected villages at the date of data collection were eligible for the study” is not readable. Why should the target populations be able to provide essential information regarding the study protocol? Living in the selected villages? The inclusion and exclusion criteria must be clearly stated.

: Thank you. The sentences have been revised.

27. Line 27, page 4: The study sample size…

: Thank you. This has been improved.

28. Line 1, page 5: “Therefore, the minimum required sample size for the study was 196 participants for each sub-population (children aged 5-15, women aged 16-59, and the elderly aged ³60).” Then you can remove the whole following sentence (However, in this study, three vulnerable groups (children aged 15 years and below, women aged 16-59, and elderly aged 60 year and over) were selected for the study, then, a total of 588 participants were required.”

: Thank you. This has been revised and improved accordingly.

29. Line 7, page 5: Did this study have a conceptual framework? If so, it must be described in the text. If not, this expression about it should be removed.

: Thank you very much for the great comment. This has been removed.

30. Lines 13-15, page 5: The complex sentence needs to be re-structured by breaking it down into a few sentences or using correct punctuations to link the sub-sentences.

: Thank you very much. The sentence has been revised and improved.

31. Lines 17-21, page 5: The long sentence should be rewrite as it is difficult to read and with grammatical errors.

: Thank you very much. This has been improved and shortened.

32. Lines 25-26, page 5: Why was substance use limited to only cigarette, alcohol, and amphetamine use? Also, how is cigarette smoking related to domestic violence?

: This is because in our literature review, it was found that substances are frequently used by Lahu people. Cigarette smoking is related to amphetamine use, and it was thus included in the study.

33. Lines 27-28, page 5: Are sexual harassment and being forced to have sex considered harm related to sex, not sexual violence?

: Thank you for the comment. We have provided considerable discussion to describe these harms. Basically, sexual violence is a sexual act or attempt to engage in a sexual act by violence or coercion and includes acts to traffic a person or acts against a person directly. This is a clear definition. However, we need to cover only sexual harassment and being forced to have sex, which were specifically found in the Lahu women. Therefore, we decided to use this term. We hope very much that you understand us.

34. Line 30, page 5: The word ‘ignorant’ may have been incorrectly used.

: I am very sorry for the mistake. Actually, the English in this article was improved by American Journal Experts (AJE) before submission. However, I agree with you that there remain many errors. Before resubmission, the article has been re-edited by AJE.

35. Lines 1, page 6: Is ‘domestic abuse’ different from ‘domestic violence?’ It is the first mention of domestic abuse in the paper. Also, please say ‘women aged 16-59’ consistently.

: I am sorry for the confusion. During the literature review, I discovered that scholars use the two terms, domestic abuse and domestic violence, interchangeably. At the time, I wanted to use all words to cover every use and put both words in the paper. However, to be consistent, “domestic abuse” has been deleted.

36. Line 4, page 6: ‘Cronbach’s alpha.’ Also, state clearly on what is the Cronbach’s alpha for? Domestic violence scale? Across the three participant groups? References to support the scales used in this study must be cited. The use of the scales (coding, recoding, cut-offs used to define the variables…), should be clearly described.

: The Cronbach alpha is used to detect the reliability of questions related to domestic among children, women, and the elderly. I have double checked and out on the numbers and reference in.

37. Several grammatical mistakes are easily found in the questionnaire development and data collection section. Please revise them carefully. For example, the following three sentences are all incorrect: did you have experienced of kicking out of the house from family member in the past year? Did you have experienced of being forced to drink alcohol, smoke, or use substances in the past year from family member? Did you have experienced of being forced to ask for money or borrow items from others?

: Thank you for the comment. I requested that AJE re-edit the manuscript. I hope that the revised version is acceptable.

38. Please make ‘statistical/data analysis’ plural (data analyses).

: Thank you. This has been improved.

39. Line 1, page 7: Please specify whether the ‘logistic regression analyses’ were bivariate, multiple, or both. The description of the variable selection for the multiple regression analysis models is hard to understand. The sentences “Some variables were controlled the effect in the model which were determined as the confounder factors for the prediction. In the final model, all significant variables and controlled variables were fitted before making interpretations” are unreadable.

: We used a binary logistic regression with both univariate and multivariate steps.

: The mentioned sentence has been revised.

40. The paragraph on the ethics also needs improvement in writing quality. Did this study need more than one protocol? If so, why? Please correct the wording – e.g., what you need from the participants was their consent, not the form. Please also correct several grammatical errors in the paragraph.

: Thank you very much. I am sorry for the many grammatical errors (even though this version was checked by AJE). The language was improved and revised again by AJE, no. 3987-18F0-6D5E-AB8E-72FF .

**Results**

All tables and narratives of the tables need to be revised.

41. Lines 19-20, page 7: Please clarify if the 646 was the number of participants recruited or included in the analyses. If the number of the participants recruited was different from that in the analyses, please provide the details of the number of participants excluded with reasons.

: The 646 participants were included in the analyses.

: No participants were excluded from the analyses.

42. Line 22-23, page 7: Please remove ‘While,’ which was incorrectly used, and change ‘elderly persons’ to ‘the elderly.’ Also, please use ‘;’ to separate full sentences, not ‘,’.

: Thank you very much. This has been improved.

43. Lines 21-24, page 7: You don’t need to repeat the number of participants in each group as it was clearly stated in the first sentence.

: Thank you. I agree with you, and this has been improved.

44. Table 1:

- Please do not use ‘.’ in titles.

: This has been removed.

- The use of ‘;’ is also incorrect.

: This has been improved.

- The table’s head is not understandable – Why was the head repeated? Please have a look at other published papers and revise them accordingly

: Thank you for the great comment. I have learned a lot from this comment.

: The table headings have been revised.

45. Line 2-5, page 9: Please correct all the sentences in this paragraph, which are unreadable and grammatically incorrect.

: All sentences were improved.

46. Table 2:

- The title is incomplete.

: This has been revised and improved.

- The contents of the table are not matched with the table’s head – while the head notes n (%) for Yes and No, one column presents number, the other presents %. Also, it’s unnecessary to present both ‘Yes’ and ‘No’ results. Let readers do the math.

: Thank you very much for the comment. You are correct. This is an important mistake and has been corrected. The column is the n and % of each group.

- While only smoking, alcohol use, and amphetamine use was mentioned in the methods, other types of substances are shown in the table. Please make them consistent. Please also include the last item, ‘Family member who used alcohol’ in the measurements in the methods.

: Thank you for the comment. All types of substance use are mentioned in questionnaire.

: ‘Family member who uses alcohol’ is now mentioned in the Method section.

47. The narrative of Table 3 also needs improvement, correcting the misuse of punctuations typos, and inconsistencies. Also, only one category should be presented in tables for binary variables.

: All the sentences have been revised.

: I am sorry if there is confusion in the presentation. Basically, we want to present the total number of participants and the number that experienced domestic violence. Domestic violence is divided into physical and mental violence. Sexual abuse is presented only for the women.

: We have also resummarized the information.

48. Line 1, page 14: Please use ‘bivariate logistic regression’ in place of ‘univariate analysis.’ Univariate analyses involve only one variable without a comparison or association and are used to describe participants’ characteristics (e.g., mean, median, %, etc.). Also, ‘smoking behavior’ is not a correct term in this context – you just asked whether they smoked (yes or no), not exploring the way they smoked.

: This section was revised again by AJE.

49. In description of relationships, please specify the direction and significance level of the associations, not just saying the variables are associated.

: Thank you. This has been improved.

50. Since the description of the regression models was not at all clear, the reader cannot understand why only religion and education were controlled for in Table 4. Same for Tables 5 and 6. Also, please use correct terminologies (e.g., multiple logistic regression analysis).

: Thank you. This has been revised and improved.

: After reviewing this point, I completely understand what you mean. The sentences have been revised and improved in all descriptions in Tables 4-6.

51. Lines 2-5, page 14: The two sentences tell the same thing and should be combined. This is also applied to Table 5 and 6.

: Thank you for the comment. All relevant sentences have now been properly combined.

52. Data in Table 4 tell clearly that the sample sizes were not calculated to represent each study population. As a result, the it was too small for children.

: You are correct. We all have learned a lot from this comment. When we work on the next project, we will carefully consider the sample size; some groups are large, while some groups are small.

: Thank you very much.

53. As commented earlier, please keep only one head for all tables.

: Thank you. All table headings have been revised.

54. The footnotes under Tables 4-6 are not understandable – why were µ values needed to tell a significance level, while p-values were already presented in the tables?

: Thank you. Based on my knowledge, the alpha value is the cut-off point, and we set the statistic to 0.05, while the p-value is obtained from the real data that can be any probability value. So, to determine the significance, we need to compare the alpha value and the p-value before deciding whether to accept or reject the hypothesis. Therefore, please consider this explanation.

**Discussion**

55. In general, the discussion section requires extensive support from an experience writer. Many statements are not understandable because of the poor quality, many others are too broad and not specifically based on the study findings. The discussions are mostly the comparisons with other studies (A supports B, A is confirmed by B), without context-specific interpretation and linkage to social policy implications.

: Thank you for such a wonderful comment. We have extensively expanded the paragraphs.

56. Lines 12-14, page 26: The sentence “The WHO [28] reported that 30.0% of domestic violence against women had different prevalence estimates in different communities that were higher than the estimates in the Lahu community in Thailand” is not at all clear.

: Thank you. The sentence has been revised.

57. Line 16, page 26: Please clarify what ‘clinical populations’ means?

: I am sorry for the unclear interpretation from the original cited paper, which mentioned people attended to in a hospital.

58. Lines 1-12, page 27: The discussion on the relationship between smoking and domestic violence relied so much on personal assumptions that are not well supported by the findings or literature.

: Thank you for the great comment. These references were replaced by the proper references.

59. Lines 13-18, page 27: The paragraph almost unreadable at all due to a poor structure and grammatical mistakes.

: Thank you. The paragraph has been revised.

60. Lines 1-2, page 28: What does ‘…domestic violence was dominated by alcohol use…’ mean? Alcohol use may be a cause of domestic violence, but how it dominates the violence is not understandable.

: You are correct that alcohol use contributes to domestic violence. The sentences have been revised.

61. Limitations are not well presented.

- The sentence does not make sense – the study’s limitations are not determined by the refusal rate.

: Thank you. I agree with you, and the sentences have been deleted.

- It’s not clear how and to what extend data collection by community health volunteers affected the data quality? What measures were taken to address the concerns? Did the volunteers receive any training? Information on data collection training should be clearly provided in the Methods.

: We did not use the community health volunteers to collect the information. We asked them to help in explaining some questions in case the participants did not clearly understand the meaning or the context of the question. This did not occur often because the participants who could not speak Thai were excluded from the study as part of the exclusion criteria. Therefore, we did not train the community participants to collect the data.

- Same for the following sentence. Please discuss clearly what were the methodological issues around the collection of sensitive data?

: Basically, as we know from our more than 10 years of experience in conducting research with Lahu people, domestic violence and other family conflicts are kept secret, and they do not tell these stories in public, particularly to individuals of a different gender. Therefore, all forms of violence are sensitive issues for them. Therefore, we used gender matching during the interview to improve the accuracy of the information.

: We have expanded the explanation in the paragraph.

62. Conclusion also need to be entirely re-written. Please avoid repeating the results or include further discussion. Instead, please summarize key findings and provide relevant recommendations that are well supported by the findings.

: Thank you very much. All of these paragraphs have been rewritten.

**References**

The reference list does not meet the journal’s requirements. Please rework on it more carefully.

: I am very sorry for the mistake and misunderstanding. I have revised all references to follow the journal guidelines from the following URL: <https://journals.plos.org/plosone/s/submission-guidelines#loc-references>.

[Note: HTML markup is below. Please do not edit.]

Reviewers' comments:

Reviewer's Responses to Questions

**Comments to the Author**

1. If the authors have adequately addressed your comments raised in a previous round of review and you feel that this manuscript is now acceptable for publication, you may indicate that here to bypass the “Comments to the Author” section, enter your conflict of interest statement in the “Confidential to Editor” section, and submit your "Accept" recommendation.

Reviewer #2: All comments have been addressed

2. Is the manuscript technically sound, and do the data support the conclusions?

The manuscript must describe a technically sound piece of scientific research with data that supports the conclusions. Experiments must have been conducted rigorously, with appropriate controls, replication, and sample sizes. The conclusions must be drawn appropriately based on the data presented.

Reviewer #2: Yes

3. Has the statistical analysis been performed appropriately and rigorously?

Reviewer #2: Yes

4. Have the authors made all data underlying the findings in their manuscript fully available?

The [PLOS Data policy](http://www.plosone.org/static/policies.action#sharing) requires authors to make all data underlying the findings described in their manuscript fully available without restriction, with rare exception (please refer to the Data Availability Statement in the manuscript PDF file). The data should be provided as part of the manuscript or its supporting information, or deposited to a public repository. For example, in addition to summary statistics, the data points behind means, medians and variance measures should be available. If there are restrictions on publicly sharing data—e.g. participant privacy or use of data from a third party—those must be specified.

Reviewer #2: Yes

5. Is the manuscript presented in an intelligible fashion and written in standard English?

PLOS ONE does not copyedit accepted manuscripts, so the language in submitted articles must be clear, correct, and unambiguous. Any typographical or grammatical errors should be corrected at revision, so please note any specific errors here.

Reviewer #2: Yes

6. Review Comments to the Author

Please use the space provided to explain your answers to the questions above. You may also include additional comments for the author, including concerns about dual publication, research ethics, or publication ethics. (Please upload your review as an attachment if it exceeds 20,000 characters)

Reviewer #2: Accept. The authors have adequately addressed the comments raised in a previous round of review. This manuscript is now acceptable for publication.

7. PLOS authors have the option to publish the peer review history of their article ([what does this mean?](https://journals.plos.org/plosone/s/editorial-and-peer-review-process#loc-peer-review-history)). If published, this will include your full peer review and any attached files.


If you choose “no”, your identity will remain anonymous but your review may still be made public.
